# Voices 2: Improving Prosodic Recognition in Schizophrenia With an Online Rehabilitation Program

**DOI:** 10.3389/fpsyg.2021.739252

**Published:** 2021-12-24

**Authors:** María Lado-Codesido, Rosa María Rey Varela, Marina Larios Quiñones, Luis Martínez Agulleiro, Julieta Ossa Basanes, María Martínez Querol, Raimundo Mateos, Carlos Spuch, Alejandro García-Caballero

**Affiliations:** ^1^University of Santiago de Compostela, Santiago de Compostela, Spain; ^2^Department of Psychiatry, Donostia University Hospital, San Sebastián, Spain; ^3^Department of Psychology, National University of Distance Education, Madrid, Spain; ^4^Centro de Rehabilitación Laboral “Nueva Vida,” Red Pública de Atención Social a Personas con Enfermedad Mental Grave y Duradera, Consejería de Políticas Sociales, Familias, Igualdad y Natalidad, Asociación Psiquiatría y Vida, Madrid, Spain; ^5^Department of Psychiatry, Complexo Hospitalario Universitario de Ferrol, Ferrol, Spain; ^6^Department of Psychiatry, University of Santiago de Compostela, Santiago de Compostela, Spain; ^7^Translational Neuroscience Research Group, Galicia Sur Health Research Institute, University of Vigo, CIBERSAM, Vigo, Spain; ^8^Department of Psychiatry, University Hospital Complex of Ourense, Ourense, Spain

**Keywords:** prosodic recognition, emotion recognition, social cognition, natural semantic metalanguage, online cognitive training, computer based cognitive training, schizophrenia, schizoaffective disorder

## Abstract

**Introduction:** Emotion recognition of voices may play an important role in interpersonal communication and patients with schizophrenia present alterations in this regard. Several on-line rehabilitation tools have been developed for treatment in this area. *Voices* is an on-line prosodic recognition program consisting of identifying different emotional tones in neutral phrases, in different sessions of gradually increasing difficulty. This training tool has previously reported benefits, and a new version has been created called *Voices 2*. The main aim of this study is to test the capacity of the *Voices 2* program to improve emotion recognition through prosody for adults with schizophrenia. Secondly, it seeks to observe durability effects 1 month after intervention.

**Method:** A randomized, single-blind, multicenter clinical trial was conducted with 44 outpatients diagnosed with schizophrenia or schizoaffective disorder. The intervention group (also called *Voices*) was treated with *Voices 2*, whereas the control group was treated with auditory training that was not related to emotions. Sociodemographic and clinical data, clinical state (PANSS), Intelligence Quotient and prosodic recognition (RMV-SV) were measured at baseline. After intervention, RMV-SV and PANSS were assessed. One month later, the RMV-SV measure was repeated.

**Results:** The control group (*n* = 19) and the *Voices* group (*n* = 22) did not differ on χ^2^, t or *U* tests in sociodemographic, clinical and psychometric variables at baseline or post-intervention (all *p*-values > 0.05). In the *Voices* group, statistically significant differences were observed in the RMV-SV scale applied post-intervention vs. that applied pre-intervention (*Z* = 2.47, *p* = 0.013). Similar results were observed in the 1-month follow-up RMV-SV vs. the pre-intervention RMV-SV (*Z* = 1.97, *p* = 0.049). PANSS scale was also assessed with no significant differences between pre vs. post measures in both groups. Lastly, *Voices 2* was rated relatively higher, based on its ease of understanding, entertainment value, usefulness and the appropriateness of use of its emotional glossary.

**Discussion:** Improvements were observed in prosodic recognition following intervention with *Voices 2* in the *Voices* group. Although these results are similar to other clinical trial rehabilitation programs, specific research on the matter remains scarce. Certain aspects, such as the durability of effects or adherence should be thoroughly studied and clarified.

**Clinical Trial Registration:** [https://doi.org/10.17605/OSF.IO/G95C4].

## Introduction

Schizophrenia is a severe and chronic mental disorder that affects 20 million people across the world (James et al., 2018). There are three types of main symptoms: positive symptoms (such as delusions, hallucinations, or disorganized speech); negative symptoms (affective flattening, alogia, or avolition) and cognitive symptoms (dysfunctions in working memory or processing speed, deficits in reasoning and abstract thinking, among others). Positive symptoms are manifested in acute episodes of the illness, while negative and cognitive symptoms are present during inter-episode phases, and they represent a significant factor in the limitation of the quality of life of patients. People with schizophrenia may present a high degree of social, occupational and academic dysfunction throughout their life (Ventura et al., 2009; Halverson et al., 2019).

The cognitive symptoms of the illness include deficits related to detection, processing and the use of social information, which enables integration and communication with other people. These tasks are part of Social Cognition (SC), and SC deficits are significantly related to deteriorating functionality in schizophrenia (Fett et al., 2011; Pinkham, 2014).

One of the bases of SC, which enables correct social interaction, is emotional processing. Emotional processing alterations do not improve with antipsychotic treatment (Penn et al., 2009), and they are related to the severity of the illness (Irani et al., 2012). Facial emotion processing deficits have been widely documented (Kohler et al., 2010; Gao et al., 2021). In studies related to other sensory channels, such as the auditory pathway, results are similar to those of facial recognition, although they are collected less frequently (Lin et al., 2018). In the auditory channel, emotional recognition is carried out through tone of voice, also called affective prosody.

Affective prosody is an important issue in the field of schizophrenia because it helps to clarify meanings and resolve ambiguity in human speech through the auditory channel, when no other channel is available (e.g., a phone call) (Paulmann and Pell, 2011). Moreover, prosodic recognition is integrated as part of the multisensory channels needed for emotion communication (composed of facial and linguistic expressions and paralinguistic inputs), and it is essential for interpersonal relations. If any of these channels fail to integrate emotional signals, interpersonal conflicts could arise (Lin et al., 2020).

Deficits in prosodic recognition are associated with negative and cognitive symptoms (Thomas et al., 2017). Basic auditory skills and auditory emotion processing are impaired in schizophrenia patients with cognitive disturbances (Kraus et al., 2019). The presence of negative symptomatology has been associated with worsening prosodic recognition (Leitman et al., 2005; Castagna et al., 2013). One of the hypotheses considered by Castagna et al. (2013) is that both the cognitive state and the negative symptoms that are altered in schizophrenia could bias the evaluation and discrimination of the auditory stimuli from the environment, making them less competitive, and therefore, more deficient than in non-pathological conditions.

Moreover, it has been shown that deficits in prosodic perception have a crucial effect on global functionality (Leitman et al., 2005, 2007). These prosodic deficits also have an impact on social skills, resulting in alterations, misunderstandings, and inappropriate social responses (Kee et al., 2003; Pinkham and Penn, 2006). Primary auditory processing has been directly related to social functioning, independently of cognition (Medalia et al., 2019).

Due to the impact of emotional processing on cognitive and social dysfunction, it is vitally important to study its treatment. Several research avenues have been developed in the treatment of prosodic recognition. Pharmacology, the use of oxytocin and neurostimulation are some of the possibilities under development, although sufficient evidence is still unavailable (Tan et al., 2018). Currently, the most effective treatment is cognitive rehabilitation (Horan and Green, 2019). According to the Cognitive Remediation Experts Workshop (Florence, Italy, April 2010), cognitive rehabilitation is defined as an intervention based on behavioral training, aimed at improving cognitive processes with the aim of achieving a durable and generalized improvement. The results of these objectives in the studies conducted are also varied. The generalized improvements observed after applying an intervention program, with regards to untrained cognitive functions, psychosocial functioning and symptomatology, have been verified in certain studies (McGurk et al., 2007; Kurtz and Richardson, 2012), although reviews point to a lack of attention to this objective (Fiszdon and Reddy, 2012). As for the durability of post-intervention effects, their short-term benefits have been demonstrated (Wykes et al., 2011; Fiszdon and Reddy, 2012; Revell et al., 2015), although research on the maintenance of these improvements in the long term continues to be limited.

Furthermore, among the cognitive rehabilitation programs published, the conditions that must be met to achieve greater effectiveness are not clear. While certain reviews support the use of computerized methods compared to other types (Byrne et al., 2015), others maintain that the type of program does not necessarily entail differences in the results (Morin and Franck, 2017). Likewise, there does not appear to be significant association between a specific intervention based on a particular cognitive function and a global intervention based on several cognitive aspects when designing an intervention program (Grant et al., 2017). Studies agree that the number of tools is scarce and the methodological quality of studies is modest (Wykes et al., 2011; Grant et al., 2017). It is necessary to further examine the design of cognitive rehabilitation tools, by conducting studies with greater methodological rigor, making it possible to compare tools and draw valid conclusions.

At present, social cognition rehabilitation programs are continuously emerging. Emotion recognition is the subdomain that presents a greater number of developed tools for rehabilitation (Fiszdon and Reddy, 2012). One of the main effective SC programs in schizophrenia is called Training Affect Recognition (TAR) (Frommann et al., 2003), which focuses on facial expression recognition of basic emotions. Another program reviewed, called Social Cognition Interaction Training (SCIT) (Penn et al., 2005; Combs et al., 2007), includes various treatment modalities and is based on emotional perception, theory of mind and social perception. The final type of intervention, named Cognitive Enhancement Treatment (CET) (Eack et al., 2007), combines aspects of SC with neurocognition training and includes a prosodic rehabilitation module.

Despite this, literature on prosodic recognition rehabilitation remains scarce. Certain programs have been designed, such as Cognitive Pragmatic Treatment (Bosco et al., 2016), which is administered based on a group therapy model, in 20 half-hour sessions. Training is based on linguistics, paralinguistics and extra-linguistics, theory of mind and other cognitive functions. The results of this study did not provide affective prosodic measurements, and the sample was too small (*n* = 17). In another type of program created by Bechi et al. (2018), based on auditory and visual training and comprising of 8 h long sessions, the results showed an improvement in auditory abilities with training and an extended benefit in the auditory channel upon completion of the visual training. Finally, SocialVille (Nahum et al., 2014) is another relevant program in prosodic recognition rehabilitation. This program combines 40 working sessions of SC exercises, including auditory perception of basic emotion training, visual affect perception, social cue perception, theory of mind, self-referential style and empathy. The results showed an improvement in SC scales and functioning (Miley et al., 2020).

In this context, the web platform www.e-motionaltraining.com was developed, which includes different rehabilitation games in SC and social skills for schizophrenia and other mental illnesses (Vázquez-Campo et al., 2016; Maroño Souto et al., 2018). A specific intervention was created on this web platform, focused on affective prosody recognition, called *Voices*. The *Voices* program consists of eight sessions of varying levels in the form of a game with 15 different trials. In each trial, sentences of speech with neutral lexical content (e.g., “*I brought what you asked for*”) are reproduced in different emotional valences (e.g., happy, angry, surprised…). These sentences are recorded by professional actors and actresses and uploaded to the program in an mp3 file. For each trial, two, three or four response options with different emotions are shown, with only one being the correct answer. Once the participant has chosen their answer, the program returns feedback regarding the correct/incorrect response. Trial difficulty increases gradually over the course of the sessions. A randomized, multicenter clinical trial was conducted, comparing the intervention with *Voices* in clinically stable patients with schizophrenia or schizoaffective disorder, compared to patients with the same diagnosis who regularly attended their routine treatment at psychosocial rehabilitation centers. The result of the prosodic recognition scale applied to evaluate the participants pre- and post-intervention was favorable for the *Voices* program, compared to conventional rehabilitation (*p* < 0.05). The participants following the *Voices* treatment filled out satisfaction surveys on the program, in which 80% of users considered the tool to be easy to use and entertaining (Lado-Codesido et al., 2019). Some of the limitations of the study include the scarcity of similar studies, hindering comparison between intervention programs, the minimal number of effective sessions and the lack of evaluation of other cognitive measures to ascertain the generalized effect of rehabilitation.

Following the initial results of the *Voices* program, the tool was optimized and adapted, creating the *Voices 2* program. New sentences were added, recorded by new voluntary actors and actresses, equipping the tool with greater diversity. A reference glossary was also created to understand certain complex emotional terms (called “emotional glossary”), which can be used by users independently. The study methodology was improved, adding an active control group and a more comprehensive post-intervention evaluation, in order to observe the durability of improvements in the short term. The *Voices 2* tool, like all the other programs on the platform www.e-motionaltraining.com, was developed in Spanish.

The main goal of this study was to assess improvements in prosodic recognition with a new version of a training tool called *Voices 2*, through a prosodic recognition scale, in clinically stable patients with schizophrenia or schizoaffective disorder. The second aim was to observe the maintenance of the benefits in a short period after intervention (1 month is proposed). The final objective was to evaluate the usability of *Voices 2* tasks from the participant’s point of view.

## Materials and Methods

**FIGURE 1:**
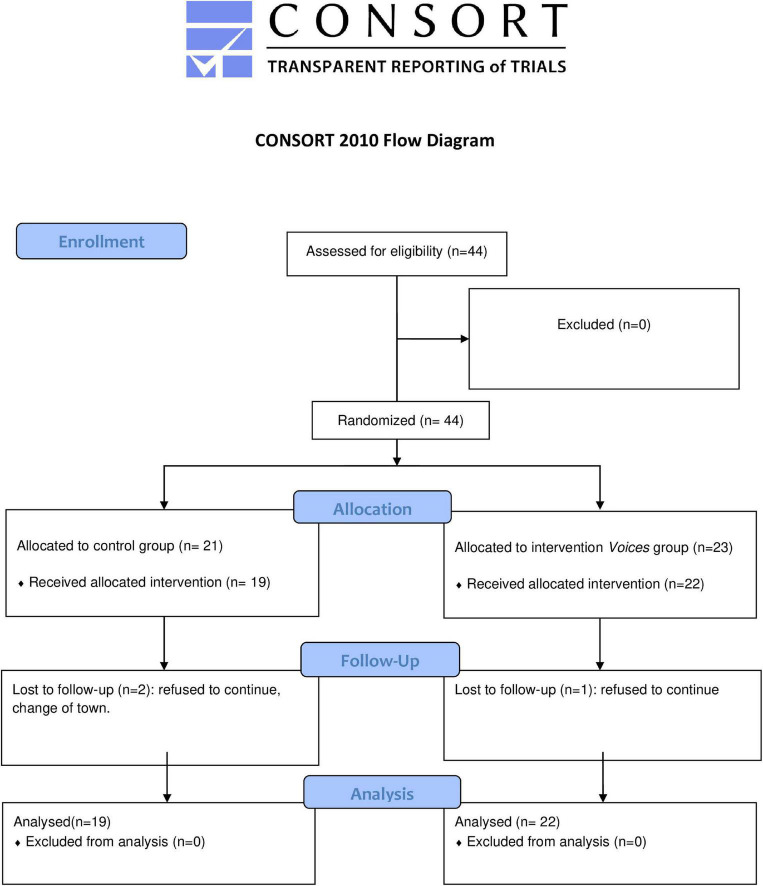
CONSORT Flowchart diagram.

A randomized, single-blind, multicenter clinical trial was conducted with 44 outpatients diagnosed with schizophrenia or schizoaffective disorder. The patients were recruited from four psychosocial rehabilitation centers in A Coruña, Madrid and Guipúzcoa. All patients attended the centers as outpatients. The selected patients were randomized by a computer-generated randomization list. The allocation sequence was randomly assigned and concealed from the research team. Three of the recruited patients were lost to follow-up, 2 in the control group (one of them refused to continue, the other moved house), and 1 in the intervention group (who refused to continue), also called the *Voices* group (Control *n* = 19, *Voices n* = 22). Therefore, the final sample was composed of 41 patients (Figure 1). The attrition rate of the intervention was 6.7%.

### Inclusion and Exclusion Criteria

We included patients who voluntarily agreed to participate in the study, who were between 18 to 65 years of age, had a diagnosis of schizophrenia or schizoaffective disorder (Diagnostic and Statistical Manual of Mental Disorders, 5th Edition), were clinically stable and followed up by the Department of Psychiatry, presented an intelligence quotient (IQ) within the normal range (> 70), who can read and write, and had no comorbidity with other psychiatric, neurological or severe auditory diseases or current substance abuse (except nicotine). We excluded patients with legal disability by reason of mental disability. During the informed consent process, the researchers confirmed that participants understood the voluntariness of their participation and the randomization strategy. Written informed consent was therefore obtained by researchers not pertaining to the clinical staff, to minimize social desirability biases. Recruitment was planned for March 2020, but it was postponed due to the COVID-19 pandemic situation from August 2020 until April 2021.

### Initial Procedures and Characterization of the Sample

Before the intervention, participants were recruited at each center. An evaluation was conducted by the research team, consisting of the collection of personal, sociodemographic and clinical data, psychometric evaluation for IQ, measured by the K-BIT test, clinical state assessed by the PANSS scale, and prosodic recognition state by the RMV-SV scale (for further information on patient evaluation tools see point 2.5). The mean age of the sample was 43.22 (SD = 1.3), most of the participants were men, and were diagnosed with schizophrenia. For more information, see Table 2.

**TABLE 1:** Description of procedure of the clinical trial.

	Before intervention	Intervention	After intervention	1-month follow-up
Description	Recruitment, selection and randomization of *Voices* and control group	8 sessions in total over a month. Each week two sessions were administered of *Voices 2*/Lyrics Training program.		
Evaluation tools	Customized datasheet RMV-SV PANSS K-BIT		RMV-SV PANSS User experience questionnaire (*Voices* group)	RMV-SV

**TABLE 2:** Demographic and clinical characteristics of the sample and by subgroups.

Variable	Group			*p*-value
	Total (*n* = 41)	Control (*n* = 19)	Voices (*n* = 22)	
Sex	Male 61% Female 39%	10 9	15 7	0.309
Age	43.22 (1.3)	43.4 (8.33)	43.(8.75)	0.890
Marital status	Single 95.1% Married 4.9%	17 2	22 0	0.209
Current cohabitation	Alone 7.3% With family 58.6% Others 34.1%	1 11 7	2 13 7	0.868
Occupation	Active 31.7% Inactive 68.3%	6 13	7 15	0.987
Education level	Primary studies 26.8% Second studies 73.2%	4 15	7 15	0.438
Diagnosis	Schizophrenia 87.8% SA disorder 12.2%	17 2	19 3	1.000
Illness duration, years	19.39 (1.4)	20.6 (10.2)	18.3 (8)	0.424
Equivalence to chlorpromazine, mg (SD)	971.76 (197.33)	1,305.9 (1,739.39)	683.1 (509.47)	0.360
PANSS	PANSS-P PANSS-N PANSS-GP PANSS-T	18.3 (8.47) 19.7 (8.7) 38.4 (15.76) 76.5 (18.3)	15.1 (7.85) 20.7 (8.8) 35.2 (16.36) 71.5 (30.1)	0.213 0.720 0.991 0.598
K-BIT	Total	98.95 (14.59)	97.41 (23.97)	0.657
	Verbal	106.00 (10.40)	105.64 (13.63)	0.925
	Non-verbal	94.89 (16.48)	91.64 (15.18)	0.514
RMV-SV		21.11 (4.1)	19.91 (4.8)	0.406

*PANSS, Positive and Negative Syndrome Scale; GP, General Psychopathology, P, Positive, N, negative, T, total. P-value between subgroups. K-BIT, Kauffman Brief Intelligence Test; RMV-SV, Reading Mind in the Voice- Spanish Version. SA disorder, schizoaffective disorder.*

After these initial procedures, participants were randomized into the *Voices* and control group. The researchers were blind to the assignment.

### Intervention Description

The control group participated using a free computerized music program called Lyrics Training^1^. This music program entails participants listening to different songs and guessing hidden words from the lyrics that are displayed on the screen while the song is playing. Each correct answer adds points to a total score that is shown at the end of the song. The difficulty of the program can be increased and adapted to the user’s ability. This program can be used autonomously, with the support of the research team where necessary. Although prosody could be considered closely connected to music, this program is focused on semantic aspects of the lyrics, and is not related to social cognitive rehabilitation, or to emotion recognition training (the real aim of the Lyrics Training program is language learning). Moreover, this program is not specific to mental illness.

The *Voices* group participated in sessions using *Voices 2*, which is the optimized version of the *Voices* program (Lado-Codesido et al., 2019). Eighty-two new trials have been added to the sixty-three initial trials, with each sentence recorded by professional actors and actresses, while new sessions of progressive difficulty have also been designed. At the start of the game, the program automatically plays a sentence with neutral lexical content, which was produced with a specific emotion, and several response options are shown with different simple and complex emotions, as explained above. Feedback is given after each trial, and when each game has been completed, the final score is displayed. For each training session, participants can play the same game level as many times as they want, and the scores of each game will be compared on the final screen. In each game, different trials appear randomly. For more specific details about the *Voices* tool, see Lado-Codesido et al. (2019).

Intervention with Lyrics Training and *Voices 2* was composed of a total of eight sessions over the course of a month. These sessions were divided into 2 weekly sessions lasting approximately 30 min. The participants attended their reference center to undergo training. A common data collection protocol was established for all centers involved in the study. Training was conducted with a personal computer or tablet for each participant, in a quiet room, with the support of trained personnel from the research team to show the participant how to use the application. Both the control and the *Voices* groups underwent treatment as usual (including drug therapy, case management and individual and group psychotherapy).

#### Natural Semantic Metalanguage

One of the problems arising from our first *Voices* clinical trial was that users showed difficulties in comprehending the lexicon referring to complex emotions. However, explaining these complex terms was not easy and dictionary definitions were, on many occasions, circular, defining one complex term with a synonym of comparable difficulty. To resolve this issue, it was necessary to seek an operative definition model avoiding circularity, i.e., a “universal” language that could disambiguate complex emotions without recurring to synonyms. From a linguistic perspective, this solution was delivered by means of the so-called “Natural Semantic Metalanguage” (NSM). The NSM is an approach to explain human emotions that allows comprehension across different cultures, previously used in other pathologies such as autism (Mullan et al., 2020) and in other fields of medicine, to facilitate effective communication with patients (Peeters and Marini, 2018). It is a “mini-language” expressed through grammar and a reduced group of “primitive concepts,” which represent the same meaning in every language (Wierzbicka, 1999). These “primitive concepts” are chosen and combined to construct a definition of emotional terms to facilitate the understanding of people with greater difficulties, such as those with psychosis. Further information can be found on the NSM webpage: https://nsm-approach.net/.

**FIGURE 2:**
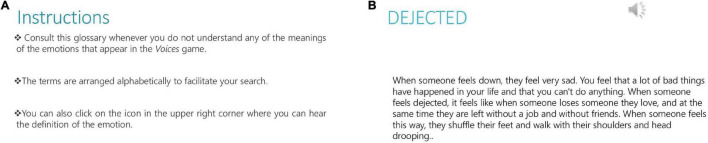
**(A)** Instructions for the emotional glossary attached to the *Voices 2* program. **(B)** Example of a complex emotional term in NSM. Reprinted from www.e-motionaltraining.com under a CC BY license, with permission from Fundación Biomédica Galicia Sur, original copyright 2018.

Specialists in NSM voluntarily collaborated in the preparation of a consultation tool called “emotional glossary.” Through NSM, they defined complex emotional terms that were used in the game, and which could pose greater difficulties to participants. This consultation tool was then made accessible from the program’s main website and participants could check it autonomously throughout the game and as many times as needed (Figure 2). Unfortunately, only two of the four centers could use the emotional glossary. The assessment questionnaire that was administered at the end of *Voices 2* intervention also asked participants to assess this tool and its ease of understanding.

### Post-testing Procedures

At the end of the intervention with *Voices 2* and Lyrics Training, both groups were retested with RMV-SV and PANSS. The *Voices* group also completed a 5-min user experience questionnaire. One month after the post-intervention test, RMV-SV was tested once more in both groups (Table 1).

### Evaluation Tools

Several tools were employed to assess the benefits of performing prosodic training, and to improve *Voices 2* tasks.

#### Patient Evaluation

All patients were assessed by the instruments described below. The results for all the scales mentioned below are provided in raw scores, except for IQ scores that are provided in standard scores:

• Customized datasheet designed by the authors for recording demographic and clinical data, including sex, age, occupation, educational level, marital status, current cohabitation, diagnosis and associated diagnoses, illness duration, handedness, and equivalence of antipsychotic treatment to chlorpromazine. The initial database characterization was obtained from this demographic data. This information was collected once at baseline, during a conventional clinical interview with the patient and based on their electronic medical history.
• *Reading the Mind in the Voices—Spanish Version, RMV-SV* (Sánchez-Reales et al., 2019). Validation of the Reading the Mind in the Voices—Test Revised (RMV-TR) scale (Golan et al., 2007), which includes 33 segments translated and adapted from English and recorded by professional actors, with four response options, with simple and complex emotions. This test was administered before, after and 1 month after finishing the intervention to both groups.
• *Positive and Negative Symptom Scale (PANSS)* (Kay et al., 1987). This scale assesses the positive and negative symptom severity. It was applied before and after intervention to both groups.
• *Kauffman Brief Intelligence Test (K-BIT)* (Kaufman and Kaufman, 2011). This test includes the measure of verbal and non-verbal intelligence in adults. It was applied before the intervention.
• User experience questionnaire, created for the evaluation of the *Voices* tool and re-adapted to the *Voices 2* tool. The questionnaire included 11 questions on a Likert-type scale (ranging from 1 = total disagreement, to 5 = total agreement) to assess different aspects of the intervention (frequency of PC or Internet use; ease of connection, ease of understanding, entertainment value, autonomy of use and usefulness of *Voices 2*; subjective perception of the benefits obtained after using the program in the usual environment, new relationships, work environment and self-esteem; duration of the intervention and assessment of the emotional glossary). It was applied post-intervention to the *Voices* group.

### Ethical Aspects

This study has been designed respecting the rules of good clinical practice and the ethical principles for medical research of the World Medical Association, which are reflected in the Declaration of Helsinki and its subsequent amendments. Likewise, European and state regulations regarding medical research are respected, particularly Organic Law 15/1999 of December 13 on the protection of personal data. All patients included were adequately informed about the purpose of the study and were asked to sign an informed consent. This study was approved by the local ethics committee (Clinical Research Ethics Committee of Galicia, Registration code: 2019/530, Euskadi, Registration code: PI2019192).

The authors confirm that all ongoing and related trials of this intervention are registered and anonymized. This study has been registered in an international registry of clinical trials, with Clinical Trial Registry Number: https://doi.org/10.17605/OSF.IO/G95C4. Under no circumstances will personal information be published or disclosed to persons outside the investigation or to the Ethics Committee for Clinical Research.

The study complies with current regulations on Intellectual Property, in accordance with Legislative Royal Decree 1/1996 of April 12. The ability of patients to understand the voluntariness of the study was assessed by researchers, by employing a clinical interview.

### Statistical Analyses

Statistical analyses were performed, using SPSS version 25.0 software. The accepted α risk was 0.05. The following tests were applied:

• A descriptive analysis was performed. The quantitative variables are presented as means (M) and standard deviation (SD) or medians (Med) and ranges. The qualitative variables are presented as frequencies and percentages.
• To compare characteristics at baseline between the control and *Voices* groups, normality of data was tested, using the Shapiro–Wilk test. All variables presented a normal distribution except for the following variables: equivalence to chlorpromazine, Total IQ, PANSS positive, general psychopathology and PANSS total at baseline. To compare two qualitative variables, the chi-squared test was used. To compare quantitative variables, Student’s *t*-test for independent samples (in Gaussian distribution) and *U*-Mann–Whitney test (in non-Gaussian distribution) were applied.
• To assess significant differences in temporal changes in RMV-SV and PANSS pre-post intervention in each group, normality was again tested with Shapiro–Wilk test. In this case, distribution was non-normal in all variables. For our purpose, Wilcoxon’s signed-rank non-parametric test was applied.
• Lastly, Cohens d statistic was obtained to estimate the effect size in the control and *Voices* groups.

## Results

A total of 44 participants were recruited and met the selected criteria. A single blinded, randomized assignation was carried out to select participants for the control group or for the *Voices* group. Three participants dropped out of the study. Finally, the statistical analysis was performed over a sample of 41 participants. All the descriptive analyses of the main variables of the sample are described in Table 2.

### Baseline Characteristics and Analysis Between Subgroups

No significant differences were found between groups in χ^2^, t or U test (all *p*-values > 0.05) in any sociodemographic or clinical measure at baseline (Table 2). RMV-SV raw mean was 19.91 (SD = 4.8) in the control group, and 21.11 (SD = 4.1) in the *Voices* group, with no statistical differences observed between groups (*t* = 0.839, *p* = 0.406). In the IQ test, no differences were observed in terms of the standard scores for total IQ (*U* = 0.445, *p* = 0.657), verbal IQ (*t* = 0.132, *p* = 0.925), or non-verbal IQ (*t* = 0.662, *p* = 0.514) between groups. Nor were any differences found in the raw PANSS scores between subgroups, confirming the homogeneity between subgroups.

### Assessment of Changes in RMV-SV Before, After and 1 Month After Intervention in Each Group

**FIGURE 3:**
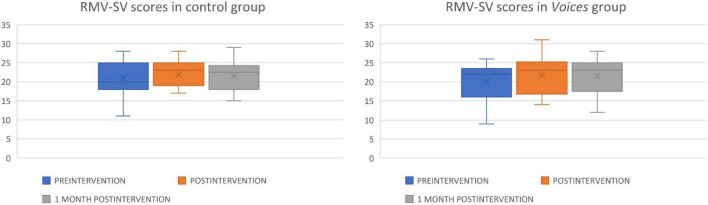
This box plot shows raw RMV-SV scores in the control and *Voices* groups before (in blue), after (in orange) and 1 month after intervention (in gray). Inside each box, “X” represents the raw RMV-SV mean, and the horizontal line shows the median of each group. Error bars represent the maximum and minimum scores of this test in each subgroup.

Raw RMV-SV scores at baseline, after intervention and 1 month after intervention in both groups are reported in Figure 3. RMV-SV scores after intervention are progressively higher compared to the previous scores, for both subgroups (control RMV-SV post-intervention *M* = 21.89, SD = 3.60, *Voices M* = 21.68, SD = 4.68). Similarly, at the 1-month follow-up, the maintenance of scores can be observed (RMV-SV 1 month follow-up control *M* = 21.44, SD = 4.01 vs. *Voices M* = 21.48, SD = 4.42). In the comparison between groups, no significant differences were observed in RMV-SV post-intervention (*t* = 0.161, *p* = 0.873) and in RMV 1-month follow-up (*t* = −0.23, *p* = 0.982).

Time (pre-post) interaction effects were investigated for raw RMV-SV scores and raw PANSS scores (Table 3). Statistically significant differences were observed in the post-intervention RMV-SV scale vs. the pre-intervention RMV-SV (*Z* = 2.47, *p* = 0.013) in the *Voices* group. Likewise, statistically significant differences were observed between the 1-month follow-up RMV-SV, compared to the pre-intervention RMV-SV (*Z* = 1.97, *p* = 0.049) in the *Voices* group. However, there were no significant differences between the post-intervention and 1-month follow-up measures in the *Voices* group. In the case of the control group, there were no significant differences when contrasting the three raw RMV-SV means. The effect size of RMV-SV between the groups was calculated (*d* = 0.26, *r* = 0.13). Effect sizes for the pre-post intervention comparisons are included in Table 3.

**TABLE 3:** RMV-SV analysis before, after and 1-month post-intervention.

	Control group				Voices group			
RMV-SV	Z	*p*-value	*d*	*r*	*Z*	*p*-value	*d*	*r*
Post vs. pre	0.727	0.467	0.20	0.09	2.472	**0.013**	0.37	0.18
1-month vs. pre	0.239	0.811	0.08	0.04	1.972	**0.049**	0.34	0.16
1-month vs. post	0.458	0.647	−0.11	−0.05	0.102	0.919	−0.04	−0.02
PANSS								
PANSS T post vs. pre	0.55	0.582	−0.04	−0.02	1.080	0.280	0.06	0.03
PANSS P post vs. pre	0.355	0.723	0.00	0.00	0.348	0.728	−0.01	−0.06
PANSS N post vs. pre	0.564	0.573	0.00	0.00	0.894	0.371	0.10	0.05
PANSS GP	0.735	0.462	−0.16	−0.08	0.996	0.319	0.00	0.00

*Post, post-intervention; Pre, preintervention; PANSS T, Total; P, Positive; N, Negative; GP, General Psychopathology; Control, control group / Voices: intervention group; RMV-SV: Reading the Mind in the Voice – Spanish Version; Z, Wilcoxon signed-rank test; d, Cohen’s d value; r, effect size.*

*In bold, p-values < 0.05 indicate statistically significative differences between post and pre-intervention scores and 1-month intervention and pre-intervention scores in Voices group.*

Furthermore, raw PANSS total and PANSS subscales scores were also assessed before and after intervention to confirm clinical stability. No significant differences were found between pre- and post-intervention measures in both groups (Table 3).

In conclusion, significant improvements were observed in RMV-SV scores after *Voices 2* intervention and these improvements were maintained 1 month later. These differences were not reported in the control group. In the case of the clinical variable PANSS, there were no temporal significant differences despite the use of the *Voices 2* program.

### User Experience Scale

**FIGURE 4:**
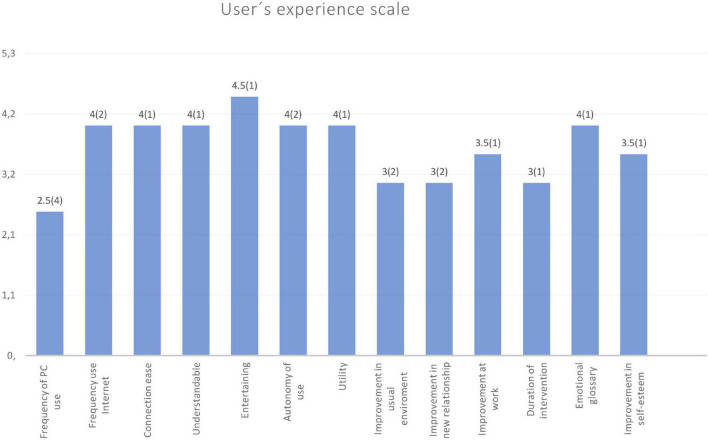
User experience scale. Medians and interquartile ranges (in brackets) are represented in each bar.

All participants in the *Voices* group completed a 5-min self-reported questionnaire to describe their perception after using the *Voices 2* program. Medians and interquartile ranges can be observed in Figure 4. It should be noted that in the scores referring to the frequencies of PC or Internet use (from “not frequent” to “very frequent”), users obtained lower medians (2.5 for PC use and four for Internet use), with wider interquartile ranges, so they are not highly familiar with this type of tool. Despite the fact that this could be an obstacle for the proper use of *Voices 2*, users rated the ease of understanding of the program (Med = 4), entertainment value (Med = 4.5), the utility of the program (Med = 4) and the convenience of the emotional glossary (Med = 4) highly.

## Discussion

In this randomized clinical study, a prosodic recognition program called *Voices 2* has been tested in patients with schizophrenia or schizoaffective disorder. A pre- and post-intervention assessment was conducted with the prosodic recognition scale RMV-SV and, in the case of clinical state, with the PANSS scale in each group. When comparing post-intervention RMV-SV between groups, no significant differences were observed. Despite this fact, the results confirmed significant differences in the post-intervention RMV-SV scale compared to the baseline values in the *Voices* group. In the control group, whose training entailed an auditory program that was not related to emotions, these results were not observed. The scores on this prosodic recognition scale also confirmed the significant differences observed 1 month after finalizing intervention in the *Voices* group, which were not observed in the control group. There were no significant differences in the clinical state of patients pre- and post-intervention in any of the subgroups. Users of the *Voices 2* program considered the tool as globally attractive and efficient. These results suggest that *Voices 2* could be useful as a specific intervention program for the rehabilitation of emotional prosody in patients with schizophrenia or schizoaffective disorder.

The results obtained in the scores of the RMV-SV are similar to the first version of the *Voices* study (Lado-Codesido et al., 2019). In the current study, it is worth noting that the improvements designed for *Voices 2* did not achieve better results compared to the first version of *Voices* (e.g., the emotional glossary, or the addition of new trials to the game). As explained above, the emotional glossary was applied in two of the four centers and, given that it was used autonomously, its impact was not strictly measured. Although participants found this tool appropriate, further research is needed to clarify its contribution. Likewise, the addition of new trials in *Voices 2* did not represent a greater improvement in results, but this variability could help avoid the repetition of phrases, thus reducing the memorization bias. The addition of an active control group in the present study implies increased methodological quality and gives the results greater rigor (Kurtz et al., 2016).

These findings are also in line with similar rehabilitation programs. For example, the SocialVille program, which we consider to be the most comparable with *Voices 2*, is based on PC games and includes specific auditory training and an active control group (Rose et al., 2015). Although significant results in the SC measures were not obtained in the initial trial (Nahum et al., 2014), when the study was repeated with a larger sample (*n* = 55), favorable results were observed for SC scales, including a prosodic identification scale (Nahum et al., 2021). At present, programs that include prosodic recognition measures, which would enable us to compare our results with other programs, continue to be extremely limited (Fiszdon and Reddy, 2012; Grant et al., 2017; Tan et al., 2018).

In this study, *Voices 2* shows the durability of the effects achieved 1 month after intervention, with results that are consistent with other rehabilitation programs. For example, SocialVille reported positive results in prosodic recognition after 6 months (Miley et al., 2020). In the review carried out by Fiszdon and Reddy (2012), 50 studies on SC treatments were reported. Six of these studies evaluate the short-term maintenance of effects (between 1 day to 6 weeks), with favorable results. Similar results can also be observed in other meta-analyses, with a low number of studies that include this variable (Grynszpan et al., 2011; Wykes et al., 2011). Other studies evaluate maintenance in the longer term (one and 2 years), such as the cognitive rehabilitation program Cognitive Enhancement Therapy (Hogarty et al., 2004; Eack et al., 2011). In a recent meta-analysis carried out by Nijman et al. (2020), it is concluded that improvements are maintained in the follow-up of studies evaluating this variable, but with lower values compared to post-treatment. These conclusions are consistent with the results of *Voices 2*. Given the significance of the durability of these improvements, as the main objective of cognitive rehabilitation, it is crucial to continue to investigate the effects in the long term, as well as the underlying neoplastic changes brought about by cognitive rehabilitation.

The scores of the satisfaction survey on *Voices 2* are also similar to those of the first version of the program, confirming the perception of this tool as accessible and attractive. Other rehabilitation programs also include this type of subjective user evaluation (Nahum et al., 2014; Rus-Calafell et al., 2014; Palumbo et al., 2017). The positive evaluation of the tool, individual motivation, the subjective perception of improvements and feedback given to users during rehabilitation could all positively contribute to treatment response and program adherence (Fiszdon et al., 2020). These points were considered when designing the *Voices 2* program.

With regards to program adherence, it is worth noting the low dropout rate of 6.7%, compared to other studies reviewed, with rates of between 23–37% (Fernandez-Gonzalo et al., 2015; Fisher et al., 2017; Grant et al., 2017; Nahum et al., 2021). This could be due to the use of *Voices 2* at the regular care centers of participants, who take part in more extensive psychiatric rehabilitation. Furthermore, *Voices 2* seeks to represent situations that are similar to the real life of patients. These two factors could be key for treatment adherence (McGurk et al., 2007, 2012; Wykes et al., 2011; Parker et al., 2013). The duration of *Voices 2* (approximately 4 weeks, compared to 8–16 weeks for the aforementioned programs), could also be associated with the differences in dropout rates.

Finally, it is worth noting the innovative creation of the emotional glossary as an exploratory tool, with the use of NSM for the first time in a rehabilitation program. In the same way as the work carried out in autism, we find this approach to be very interesting, as a new avenue for the lexical understanding of emotional terms in populations that present deficits in emotional processing. Furthermore, such tools facilitate the autonomous use of the *Voices 2* program. Despite the fact that it has not been possible to use the glossary at all centers, users defined it as appropriate (Med = 4). After this first experience with NSM, we feel it would be interesting to expand on and further the study of the emotional glossary for future versions of the program.

## Limitations

Certain limitations have been observed in this study. It is necessary to give cognitive rehabilitation studies sufficient methodological quality to enable comparison between studies. According to the criteria of the Clinical Trial Assessment Measure (CTAM), a larger clinical sample would be necessary. The small effect size is also a limitation. Moreover, repeating the RMV-SV scale on three different occasions could represent a learning bias, although this is not in line with the differences observed between subgroups. The repeated use of the RMV-SV is related to the lack of similar measures in Spanish, as well as its ease of application, without the need for prior training. In addition, the use of actors and actresses for play-acting vocal emotions could be considered an artificial way of expressing emotions (Jürgens et al., 2011), and it does not represent natural conditions. Furthermore, following the objectives of cognitive rehabilitation, the inclusion of measures of other cognitive or psychosocial functioning aspects in this study could make it possible to clarify whether the effects of *Voices 2* are generalized, an objective that has not been investigated in this study.

## Conclusion

*Voices 2*, the on-line prosodic recognition rehabilitation program, could be effective in improving prosodic recognition in patients with schizophrenia or schizoaffective disorder, as well as being described as attractive and efficient from the user’s perspective. The durability of the improvements observed is maintained 1 month after intervention with *Voices 2*. These results support the use of *Voices 2* and promote the progress of aspects of psychiatric rehabilitation that still need to be developed. This new tool could provide benefits in terms of the interpersonal communication of patients with schizophrenia, and changes in prosodic recognition in the short term. Further studies could be conducted to continue to examine knowledge of prosodic rehabilitation, improving the quality of studies on emotion recognition intervention and promoting the personalization of rehabilitation treatment for schizophrenia.

## Data Availability Statement

The datasets presented in this study can be found in online repositories. The names of the repository/repositories and accession number(s) can be found below: https://doi.org/10.17605/OSF.IO/G95C4.

## Ethics Statement

The studies involving human participants were reviewed and approved by Clinical Research Ethics Committee of Galicia, Registration code: 2019/530, Euskadi, Registration code: PI2019192. The patients/participants provided their written informed consent to participate in this study.

## Author Contributions

AG-C and ML-C created the Voices 2 program, selected the participants, applied the intervention, extracted data, and supervised the study. AG-C, ML-C, and LM designed the study. ML-C, ML, RR, LM, JO, and MM participated in patient selection and obtained and extracted data. ML-C, AG-C, and RM wrote and revised the manuscript. All authors have had full access to the study data, have personally reviewed the manuscript and have given their final approval to the version attached.

## Conflict of Interest

The authors declare that the research was conducted in the absence of any commercial or financial relationships that could be construed as a potential conflict of interest.

## Publisher’s Note

All claims expressed in this article are solely those of the authors and do not necessarily represent those of their affiliated organizations, or those of the publisher, the editors and the reviewers. Any product that may be evaluated in this article, or claim that may be made by its manufacturer, is not guaranteed or endorsed by the publisher.

## Footnotes

1
www.lyricstrainning.com

## References

[B1] Bechi M. Bosia M. Spangaro M. Pigoni A. Buonocore M. Scrofani D. (2018). Visual and audio emotion processing training for outpatients with schizophrenia: an integrated multisensory approach. *Neuropsychol. Rehabil.* 28 1131–1144. 10.1080/09602011.2016.1240698 27712400

[B2] Bosco F. M. Gabbatore I. Gastaldo L. Sacco K. (2016). Communicative-Pragmatic Treatment in Schizophrenia: A Pilot Study. *Front. Psychol.* 7:1–12. 10.3389/fpsyg.2016.00166 26941667 PMC4762993

[B3] Byrne L. K. Pan L. Mccabe M. Mellor D. Xu Y. (2015). Assessment of a six-week computer-based remediation program for social cognition in chronic schizophrenia. *Shanghai Arch. Psychiatry* 27 296–306. 10.11919/j.issn.1002-0829.215095 26977127 PMC4764004

[B4] Castagna F. Montemagni C. Maria Milani A. Rocca G. Rocca P. Casacchia M. (2013). Prosody recognition and audiovisual emotion matching in schizophrenia: The contribution of cognition and psychopathology. *Psychiatry Res.* 205 192–198. 10.1016/j.psychres.2012.08.038 22985542

[B5] Combs D. R. Adams S. D. Penn D. L. Roberts D. Tiegreen J. Stem P. (2007). Social Cognition and Interaction Training (SCIT) for inpatients with schizophrenia spectrum disorders: Preliminary findings. *Schizophr. Res.* 91 112–116. 10.1016/j.schres.2006.12.010 17293083

[B6] Eack S. M. Greenwald D. Hogarty S. S. Keshavan M. S. (2011). One-Year Durability of the Effects of Cognitive Enhancement Therapy on Functional Outcome in Early Schizophrenia. *Schizophr. Res.* 120 210–216. 10.1016/j.schres.2010.03.042.One-Year PMC2900399 20472402

[B7] Eack S. M. Hogarty G. E. Greenwald D. P. Hogarty S. S. Keshavan M. S. (2007). Cognitive enhancement therapy improves emotional intelligence in early course schizophrenia: Preliminary effects. *Schizophr. Res.* 89 308–311. 10.1016/j.schres.2006.08.018 17055227 PMC2921636

[B8] Fernandez-Gonzalo S. Turon M. Jodar M. Pousa E. Hernandez Rambla C. García R. (2015). A new computerized cognitive and social cognition training specifically designed for patients with schizophrenia/schizoaffective disorder in early stages of illness: A pilot study. *Psychiatry Res.* 228 501–509. 10.1016/j.psychres.2015.06.007 26163731

[B9] Fett A.-K. J. Viechtbauer W. Dominguez M.-G. Penn D. L. van Os J. Krabbendam L. (2011). The relationship between neurocognition and social cognition with functional outcomes in schizophrenia: A meta-analysis. *Neurosci. Biobehav. Rev.* 35 573–588. 10.1016/j.neubiorev.2010.07.001 20620163

[B10] Fisher M. Nahum M. Howard E. Rowlands A. Brandrett B. Kermott A. (2017). Supplementing intensive targeted computerized cognitive training with social cognitive exercises for people with schizophrenia: An interim report. *Psychiatr. Rehabil. J.* 40 21–32. 10.1037/prj0000244 28368179 PMC5380146

[B11] Fiszdon J. M. Reddy L. F. (2012). Review of social cognitive treatments for psychosis. *Clin. Psychol. Rev.* 32 724–740. 10.1016/j.cpr.2012.09.003 23059624

[B12] Fiszdon J. M. Kurtz M. M. Parente L. Choi J. Connecticut V. A. System H. (2020). What variables predict cognitive remediation associated improvement in individuals with psychosis? *Schizophr. Res. Cogn.* 19:100148. 10.1016/j.scog.2019.100148 31832338 PMC6889739

[B13] Frommann N. Streit M. Wölwer W. (2003). Remediation of facial affect recognition impairments in patients with schizophrenia: a new training program. *Psychiatry Res.* 117 281–284. 10.1016/S0165-1781(03)00039-8 12686371

[B14] Gao Z. Zhao W. Liu S. Liu Z. Yang C. Xu Y. (2021). Facial Emotion Recognition in Schizophrenia. *Front. Psychiatry* 12:1–10. 10.3389/fpsyt.2021.633717 34017272 PMC8129182

[B15] Golan O. Baron-Cohen S. Hill J. J. Rutherford M. D. (2007). The ‘Reading the Mind in the Voice’ Test-Revised: A Study of Complex Emotion Recognition in Adults with and Without Autism Spectrum Conditions. *J. Autism Dev. Disord.* 37 1096–1106. 10.1007/s10803-006-0252-5 17072749

[B16] Grant N. Lawrence M. Preti A. Wykes T. Cella M. (2017). Social cognition interventions for people with schizophrenia: a systematic review focussing on methodological quality and intervention modality. *Clin. Psychol. Rev.* 56 55–64. 10.1016/j.cpr.2017.06.001 28688282

[B17] Grynszpan O. Perbal S. Pelissolo A. Fossati P. Jouvent R. Dubal S. (2011). Efficacy and specificity of computer-assisted cognitive remediation in schizophrenia: A meta-analytical study. *Psychol. Med.* 41 163–173. 10.1017/S0033291710000607 20380784

[B18] Halverson T. F. Orleans-Pobee M. Merritt C. Sheeran P. Fett A. Penn D. L. (2019). Pathways to functional outcomes in schizophrenia spectrum disorders: Meta-analysis of social cognitive and neurocognitive predictors. *Neurosci. Biobehav. Rev.* 105 212–219. 10.1016/j.neubiorev.2019.07.020 31415864

[B19] Hogarty G. E. Flesher S. Ulrich R. Carter M. Greenwald D. Pogue-Geile M. (2004). Cognitive Enhancement Therapy for Schizophrenia. *Arch. Gen. Psychiatry* 61 866–876. 15351765 10.1001/archpsyc.61.9.866

[B20] Horan W. P. Green M. F. (2019). Treatment of social cognition in schizophrenia: Current status and future directions. *Schizophr. Res.* 203 3–11. 10.1016/j.schres.2017.07.013 28712968

[B21] Irani F. Seligman S. Kamath V. Kohler C. Gur R. C. (2012). A Meta-Analysis of Emotion Perception and Functional Outcomes in Schizophrenia. *Schizophr. Res.* 137 203–211. 10.1016/j.schres.2012.01.023.A 22341200 PMC3351501

[B22] James S. L. Abate D. Abate K. H. Abay S. M. Abbafati C. Abbasi N. (2018). Global, regional, and national incidence, prevalence, and years lived with disability for 354 diseases and injuries for 195 countries and territories, 1990–2017: a systematic analysis for the Global Burden of Disease Study 2017. *Lancet* 392 1789–1858. 10.1016/S0140-6736(18)32279-7 30496104 PMC6227754

[B23] Jürgens R. Hammerschmidt K. Fischer J. (2011). Authentic and Play-Acted Vocal Emotion Expressions Reveal Acoustic Differences. *Front. Psychol.* 2:1–11. 10.3389/fpsyg.2011.00180 21847385 PMC3148714

[B24] Kaufman A. Kaufman N. (2011). *K-BIT: Test Breve de Inteligencia de Kaufman.* London: Pearson.

[B25] Kay S. R. Fiszbein A. Opfer L. A. (1987). The Positive and Negative Syndrome Scale (PANSS) for Schizophrenia. *Schizophr. Bull.* 13 261–276. 10.1093/schbul/13.2.261 3616518

[B26] Kee K. S. Green M. F. Mintz J. Brekke J. S. (2003). Is emotion processing a predictor of functional outcome in schizophrenia? *Schizophr. Bull.* 29, 487–497. 10.1093/oxfordjournals.schbul.a007021 14609242

[B27] Kohler C. G. Walker J. B. Martin E. A. Healey K. M. Moberg P. J. (2010). Facial Emotion Perception in Schizophrenia: A Meta-analytic Review. *Schizophr. Bull.* 36 1009–1019. 10.1093/schbul/sbn192 19329561 PMC2930336

[B28] Kraus M. S. Walker T. M. Jarskog L. F. Millet R. A. Keefe R. S. E. (2019). Basic auditory processing de fi cits and their association with auditory emotion recognition in schizophrenia ✩. *Schizophr. Res.* 204 155–161. 10.1016/j.schres.2018.08.031 30268821

[B29] Kurtz M. M. Richardson C. L. (2012). Social cognitive training for schizophrenia: A meta-analytic investigation of controlled research. *Schizophr. Bull.* 38 1092–1104. 10.1093/schbul/sbr036 21525166 PMC3446217

[B30] Kurtz M. M. Gagen E. Rocha N. B. F. Machado S. Penn D. L. (2016). Comprehensive treatments for social cognitive deficits in schizophrenia: A critical review and effect-size analysis of controlled studies. *Clin. Psychol. Rev.* 43 80–89. 10.1016/j.cpr.2015.09.003 26437567

[B31] Lado-Codesido M. Méndez Pérez C. Mateos R. Olivares J. M. García Caballero A. (2019). Improving emotion recognition in schizophrenia with “VOICES”: An on-line prosodic self-training. *PLoS One* 14:e0210816. 10.1371/journal.pone.0210816 30682067 PMC6347191

[B32] Leitman D. I. Foxe J. J. Butler P. D. Saperstein A. Revheim N. Javitt D. C. (2005). Sensory contributions to impaired prosodic processing in schizophrenia. *Biol. Psychiatry* 58 56–61. 10.1016/j.biopsych.2005.02.034 15992523

[B33] Leitman D. I. Hoptman M. J. Foxe J. J. Saccente E. Wylie G. R. Nierenberg J. (2007). The neural substrates of impaired prosodic detection in schizophrenia and its sensorial antecedents. *Am. J. Psychiatry* 164, 474–482. 10.1176/appi.ajp.164.3.474 17329473

[B34] Lin Y. Ding H. Zhang Y. (2018). Emotional prosody processing in schizophrenic patients: A selective review and meta-analysis. *J. Clin. Med.* 7:363. 10.3390/jcm7100363 30336573 PMC6210777

[B35] Lin Y. Ding H. Zhang Y. (2020). Multisensory Integration of Emotion in Schizophrenic Patients. *Multisens. Res.* 33 865–901. 10.1163/22134808-bja10016 33706267

[B36] Maroño Souto Y. Vázquez Campo M. Díaz Llenderrozas F. Rodríguez, Álvarez M. Mateos R. (2018). Randomized Clinical Trial with e-MotionalTraining^®^ 1.0 for Social Cognition Rehabilitation in Schizophrenia. *Front. Psychiatry* 9:1–9. 10.3389/fpsyt.2018.00040 29535646 PMC5834490

[B37] McGurk S. R. Eack S. M. Kurtz M. Mueser K. T. (2012). Cognitive Remediation and Psychosocial Rehabilitation for Individuals with Severe Mental Illness. *Rehabil. Res. Pract.* 2012 1–2. 10.1155/2012/283602 23320176 PMC3540926

[B38] McGurk S. R. Twamley E. W. Sitzer D. I. McHugo G. J. Mueser K. T. (2007). Reviews and Overviews A Meta-Analysis of Cognitive Remediation in Schizophrenia. *Am. J. Psychiatry* 164 1791–1802. 18056233 10.1176/appi.ajp.2007.07060906 PMC3634703

[B39] Medalia A. Saperstein A. M. Qian M. Javitt D. C. (2019). Impact of baseline early auditory processing on response to cognitive remediation for schizophrenia. *Schizophr. Res.* 208, 397–405. 10.1016/j.schres.2019.01.012 30665714 PMC6739117

[B40] Miley K. Fisher M. Nahum M. Howard E. Rowlands A. Brandrett B. (2020). Six month durability of targeted cognitive training supplemented with social cognition exercises in schizophrenia. *Schizophr. Res. Cogn.* 20:100171. 10.1016/j.scog.2019.100171 31908976 PMC6938953

[B41] Morin L. Franck N. (2017). Rehabilitation interventions to promote recovery from schizophrenia: A systematic review. *Front. Psychiatry* 8:100. 10.3389/fpsyt.2017.00100 28659832 PMC5467004

[B42] Mullan K. Peeters B. Sadow L. (2020). “Using Minimal English to Model a Parental Understanding of Autism,” in *Studies in Ethnopragmatics, Cultural Semantics, and Intercultural Communication: Ethnopragmatics and Semantic Analysis*, eds Mullan K. Peeters B. Sadow L. (Singapore: Springer Singapore), 1–256. 10.1007/978-981-32-9983-2

[B43] Nahum M. Fisher M. Loewy R. Poelke G. Ventura J. Nuechterlein K. H. (2014). A novel, online social cognitive training program for young adults with schizophrenia: A pilot study. *Schizophr. Res. Cogn.* 1 e11–e19. 10.1016/j.scog.2014.01.003 25267937 PMC4175473

[B44] Nahum M. Lee H. Fisher M. Green M. F. Hooker C. I. Ventura J. (2021). Online Social Cognition Training in Schizophrenia: A Double-Blind, Randomized, Controlled Multi-Site Clinical Trial. *Schizophr. Bull.* 47 108–117. 10.1093/schbul/sbaa085 32614046 PMC7825077

[B45] Nijman S. A. Veling W. van der Stouwe E. C. D. Pijnenborg G. H. M. (2020). Social Cognition Training for People With a Psychotic Disorder: A Network Meta-analysis. *Schizophr. Bull.* 46 1086–1103. 10.1093/schbul/sbaa023 32162658 PMC7505203

[B46] Palumbo D. Mucci A. Piegari G. D’Alise V. Mazza A. Galderisi S. (2017). SoCIAL – training cognition in schizophrenia: A pilot study. *Neuropsychiatr. Dis. Treat.* 13 1947–1956. 10.2147/NDT.S136732 28790830 PMC5530058

[B47] Parker S. Foley S. Walker P. Dark F. (2013). Improving the social cognitive deficits of schizophrenia: a community trial of Social Cognition and Interaction Training (SCIT). *Australas. Psychiatry* 21 346–351. 10.1177/1039856213486305 23671226

[B48] Paulmann S. Pell M. D. (2011). Is there an advantage for recognizing multi-modal emotional stimuli? *Motiv. Emot.* 35 192–201. 10.1007/s11031-011-9206-0

[B49] Peeters B. Marini M. (2018). “Narrative Medicine Across Languages and Cultures: Using Minimal English for Increased Comparability of Patients’ Narratives,” in *Minimal English for a Global World: Improved Communication Using Fewer Words*, ed. Goddard C. (London: Palgrave Macmillan), 259–286. 10.1007/978-3-319-62512-6

[B50] Penn D. L. Keefe R. S. E. Davis S. M. Meyer P. S. Perkins D. O. Losardo D. (2009). The effects of antipsychotic medications on emotion perception in patients with chronic schizophrenia in the CATIE trial. *Schizophr. Res.* 115 17–23. 10.1016/j.schres.2009.08.016 19766459 PMC2765056

[B51] Penn D. Roberts D. L. Munt E. D. Silverstein E. Jones N. Sheitman B. (2005). A pilot study of social cognition and interaction training (SCIT) for schizophrenia. *Schizophr. Res.* 80 357–359. 10.1016/j.schres.2005.07.011 16139480

[B52] Pinkham A. E. (2014). Social Cognition in Schizophrenia. *J. Clin. Psychiatry* 75 14–19. 10.4088/JCP.13065su1.04 24919166

[B53] Pinkham A. E. Penn D. L. (2006). Neurocognitive and social cognitive predictors of interpersonal skill in schizophrenia. *Psychiatry Res*. 143, 167–178. 10.1016/j.psychres.2005.09.005 16859754

[B54] Revell E. R. Neill J. C. Harte M. Khan Z. Drake R. J. (2015). A systematic review and meta-analysis of cognitive remediation in early schizophrenia. *Schizophr. Res.* 168 213–222. 10.1016/j.schres.2015.08.017 26305063

[B55] Rose A. Vinogradov S. Fisher M. Green M. F. Ventura J. Hooker C. (2015). Randomized controlled trial of computer-based treatment of social cognition in schizophrenia: the TRuSST trial protocol. *BMC Psychiatry* 15:142. 10.1186/s12888-015-0510-1 26138715 PMC4489025

[B56] Rus-Calafell M. Gutiérrez-Maldonado J. Ribas-Sabaté J. (2014). A virtual reality-integrated program for improving social skills in patients with schizophrenia: A pilot study. *J. Behav. Ther. Exp. Psychiatry* 45 81–89. 10.1016/j.jbtep.2013.09.002 24063993

[B57] Sánchez-Reales S. Caballero-Peláez C. Prado-Abril J. Inchausti F. Lado-Codesido M. García-Caballero A. (2019). Spanish validation of the “Reading the Mind in the Voice” task: A study of complex emotion recognition in adults with autism spectrum conditions. *Res. Autism Spectr. Disord.* 67:101421. 10.1016/j.rasd.2019.101421

[B58] Tan B. Lee S. Lee J. (2018). Social cognitive interventions for people with schizophrenia: A systematic review. *Asian J. Psychiatr.* 35 115–131. 10.1016/j.ajp.2016.06.013 27670776

[B59] Thomas M. L. Green M. F. Hellemann G. Sugar C. A. Tarasenko M. Calkins M. E. (2017). Modeling Deficits From Early Auditory Information Processing to Psychosocial Functioning in Schizophrenia. *JAMA Psychiatry* 74:37. 10.1001/jamapsychiatry.2016.2980 27926742 PMC5453308

[B60] Vázquez-Campo M. Maroño Y. Lahera G. Mateos R. García-Caballero A. (2016). e-Motional Training^®^ : Pilot study on a novel online training program on social cognition for patients with schizophrenia. *Schizophr. Res. Cogn.* 4 10–17. 10.1016/j.scog.2015.11.007 28740808 PMC5506727

[B61] Ventura J. Hellemann G. S. Thames A. D. Koellner V. Nuechterlein K. H. (2009). Symptoms as mediators of the relationship between neurocognition and functional outcome in schizophrenia: A meta-analysis. *Schizophr. Res.* 113 189–199. 10.1016/j.schres.2009.03.035 19628375 PMC2825750

[B62] Wierzbicka A. (1999). *Emotions across Languages and Cultures: Diversity and Universals*, 1st Edn. Cambrigde: Cambrigde University Press.

[B63] Wykes T. Huddy V. Cellard C. McGurk S. R. Czobor P. (2011). A Meta-Analysis of Cognitive Remediation for Schizophrenia: Methodology and Effect Sizes. *Am. J. Psychiatry* 168 472–485. 10.1176/appi.ajp.2010.10060855 21406461

